# The validity of three neo-classical facial canons in young adults originating from the Arabian Peninsula

**DOI:** 10.1186/s13005-015-0064-y

**Published:** 2015-03-13

**Authors:** Maisa O Al-Sebaei

**Affiliations:** Department of Oral and Maxillofacial Surgery, King AbdulAziz University, Faculty of Dentistry, PO Box 80209, Jeddah, 21589 Kingdom of Saudi Arabia

**Keywords:** Neo-classical canons, Facial anthropometry, Direct anthropometry, Facial proportions

## Abstract

**Introduction:**

Understanding facial harmony and proportions is essential for facial reconstructive procedures and orthognathic surgery planning. In the literature, the neoclassical facial canons have been revisited in populations including North American whites and African Americans. The purpose of this study was to establish a baseline for selected facial anthropometric measurements and test the validity of 3 neoclassical facial canons in a cohort of young Saudi adults originating from the Arabian Peninsula.

**Methods:**

The study group consisted of 168 healthy, esthetically pleasing Saudi Arabian dental students originating from the Arabian Peninsula (93 males and 75 females, age 20–24 years). Using a caliper, three neoclassical facial canons were measured; the vertical thirds of the face, the orbital canon (intercanthal distance = eye fissure length), and the orbito-nasal canon (intercanthal distance = nasal width) and analyzed using Student’s t-test, general linear modeling, and pairwise comparison of means.

**Results:**

The upper, middle, and lower thirds were not equal in measurement to each other (*p* < 0.0001). Sex dimorphism was observed in the lower facial third and nasal width measurements, with both larger in men (both p < 0.0001). The majority of subjects had longer upper and lower thirds than middle thirds, with 91.4% of males and 88% of females demonstrating a larger lower third than middle third. The most frequent variation in the orbital canon was a wider intercanthal distance than eye fissure length (55.9% of males and 74.7% of females). The most frequent variation in the orbito-nasal canon was a wider nasal width than intercanthal distance (92% of males and 56% of females).

**Conclusions:**

Although these individuals are esthetically pleasing, they do not exhibit equal facial thirds or conform to orbital or orbito-nasal canons. The three neoclassical canons studied could not be validated in young adults originating from the Arabian Peninsula. Thus, the esthetic goals in reconstructive and orthognathic surgery should respect this ethnic variation.

## Introduction

The human sculptures created in ancient Greece were derived from proportions that followed established rules or so called “canons” [1]. These canons are based on the hypothesis that in a harmonious face, certain fixed ratios exist between different parameters. Leonardo da Vinci described the body and facial canons in the late 1400s. This work was followed by Albrecht Dürer in the 1500s, who defined the three equal lengths of the face (the forehead, the nose, and the mouth and chin), as well as the intercanthal distance being equal to the eye fissure length [2]. These neoclassical facial canons can be regarded as precursors to the current anthropometric facial indices, which were used by anatomists, medical artists, maxillofacial and esthetic surgeons, orthodontists, and esthetic dentists [3-11].

In the twentieth century, orthodontists continued to define facial proportions through the popularization of cephalometrics (an indirect method of anthropometry) [12]. It was not until the 1980s that Leslie Farkas, the father of modern facial anthropometry, revisited the classic cannons for facial proportions as he measured and compared the neoclassical canons in different ethnicities and craniofacial deformities such as clefts [10,13-15]. The validity of these canons was rejected in the races studied, with only a minor percentage of the studied population actually exhibiting the neoclassical canons [16].

Evaluation of facial esthetics is essential during treatment planning of prosthodontic, orthodontic, plastic facial reconstructive surgery, and orthognathic surgery. Several textbooks and journal articles use derivatives of neoclassical canons such as the facial thirds, where the face is divided vertically into three regions of equivalent height, which is used instead of the facial three-section canon. Additionally, the rule of fifths, which divides the face in the transverse dimension into five equal parts by assuming that the intercanthal distance is equal to the nasal width and widths of the eyes, incorporates orbital and orbito-nasal canons [17,18].

Thus far, no data have been published on the validity of neoclassical facial canons in a Saudi Arabian population. Therefore, this study aimed to determine the validity of the neoclassical canons for young adults in Saudi Arabia originating from the Arabian Peninsula, as well as to establish a baseline for the norms and explore sexual dimorphism in this population.

## Methods

This study was approved by the Research Ethics Committee of the Faculty of Dentistry (REC-FD, King AbdulAziz Faculty of Dentistry, Jeddah, Saudi Arabia). Participation in the study was voluntary and each subject signed a consent form explaining the procedure.

### Subjects

The study group consisted of 168 healthy, esthetically pleasing Saudi Arabians: 93 males and 75 females ranging in age from 20 to 24 years old. All subjects were dental students at King AbdulAziz University, Jeddah.

The following criteria were used to determine if the subject was “esthetically pleasing”: (1) Angle class I molar and canine relationship, (2) mild convex to straight profile on clinical examination (3) normal growth and development, and (4) no obvious craniofacial or dentofacial deformities. The inclusion criteria were as follows: (1) esthetically pleasing as defined above; (2) all teeth present and erupted into occlusion excluding the third molars; and (3) the subject’s parents and maternal and paternal grandparents all originating from the Arabian Peninsula. The exclusion criteria consisted of (1) previous history of orthodontic treatment and (2) previous history of any cosmetic, reconstructive, or corrective facial surgery.

### Facial measurements

A sliding caliber (Seritex, Inc. Tinton Falls, NJ) was used to measure selected anthropometric facial components directly on each study subject. Measurements were performed in accordance with the well-established methods published by Farkas [19]. All measurements were collected by the author (MOS) with the subject’s head in the neutral position and recorded in millimeters. To evaluate reproducibility of the reading, an intra-reliability test was performed. Ten subjects were selected at random and their measurements were recorded at two different times, two weeks apart. A kappa test indicated significant agreement between both recorded measurements, with kappa = 0.783, p < 0.001.

The following measurements were assessed:

#### Vertical canon

**Figure 1:**
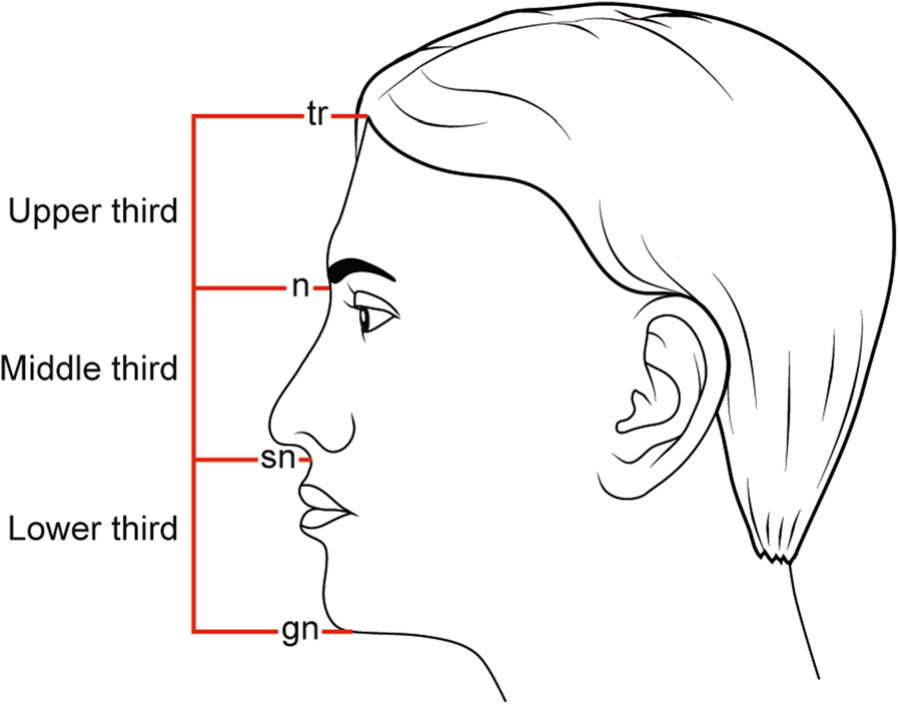
**The vertical canon: the face is divided into three equal sections.** ***tr: trichion, n: nasion, sn: subnasale, and gn: gnathion.***

The face is divided into equal thirds by a horizontal line passing through the trichion, nasion, subnasale, and gnathion (*tr-n = n-sn = sn-gn*) (Figure 1).

- Upper facial third: trichion to nasion (*tr-n*).
- Middle facial third: nasion to subnasale (*n-sn*).
- Lower facial third: subnasale to gnathion (*sn-gn*).

#### Horizontal canon 1 (orbital canon)

**Figure 2:**
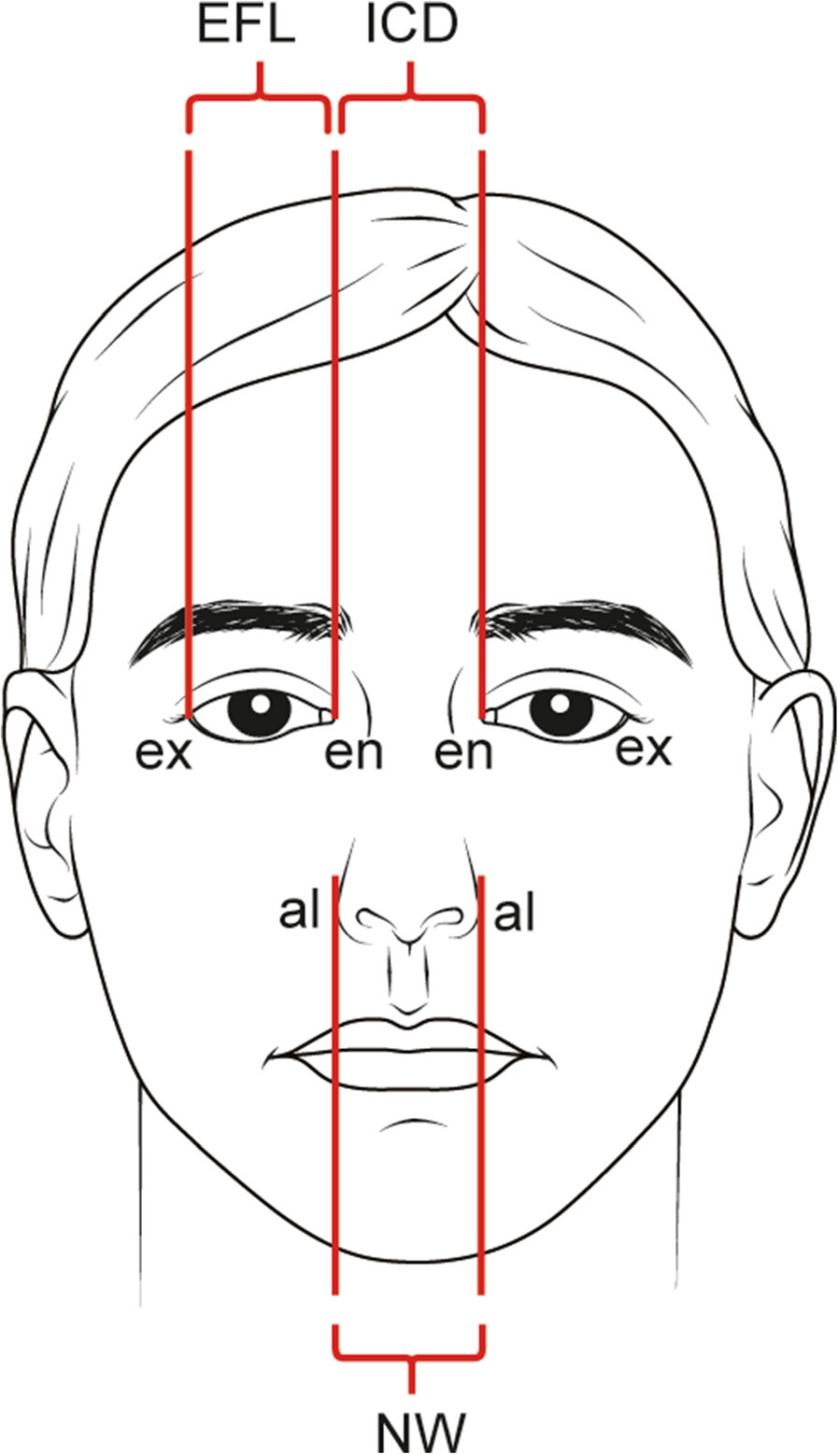
**Two horizontal canons: orbital and orbito-nasal** ***: EFL: eye fissure length, ICD: intercanthal distance, NW: nasal width, ex: exocanthion, en: endocanthion, al: alare.***

The intercanthal distance (ICD) equals the width of the eye or eye fissure length (EFL) (*ex-en = en-en*) (Figure 2).

- EFL: exocanthion to endocanthion (*ex-en*).
- ICD: endocanthion to endocanthion (*en-en).*

#### Horizontal canon 2 (Orbito-nasal canon)

The ICD equals the nasal width (NW) (*en-en = al-al*) (Figure 2).

- ICD: endocanthion to endocanthion (*en-en).*
- NW: alare to alare (*al-al*).

### Data analysis

The data were entered into a datasheet and analyzed using the SAS package (version 9.2, Cary, N.C). Values were expressed as means ± standard deviation (SD). The level of significance was set at *p* < 0.05.

A Student’s t-test was used to compare the male and female mean measurements. A general linear model test (GLM) was used to compare the measurements of the vertical canons overall and according to sex. A pairwise comparison of mean measurements was performed using the least significant difference test separately for each sex.

A facial canon was accepted as *equal* if the difference between the measurements was 0–1 mm [10,20]. The chi-square test was used to compare sexes with regard to the vertical canon (*tr-n = n-sn, n-sn = sn-gn and tr-n = sn-gn*), orbital canon (*ex-en = en-en*), and orbito-nasal canon (*en-en = al-al*). The level of significance was set at *p* < 0.05.

## Results

The mean measurements for the vertical and horizontal canons are shown in Table 1. When comparing the three measurements of the vertical canon of the face in all subjects (both sexes combined), the upper, middle, and lower thirds were not equal to each other; instead they significantly differed (*p* < 0.0001). Similarly, both the orbital and orbito-nasal canon measurements were significantly different from each other (both *p* < 0.001).

**Table 1:** **Mean measurements for males and females and comparison between sexes**

**Measurement**	**Males N = 75**	**Females N = 93**	***p*** **value**
**Upper third**	64.71 (7.51)	65.46 (6.90)	0.51
**Middle third**	54.12 (4.34)	53.19 (4.13)	0.16
**Lower third**	65.02 (5.16)	60.27 (4.62)	<0.0001*
**Eye fissure length**	32.85 (2.73)	32.41 (3.44)	0.37
**Inter-canthal distance**	30.30 (3.10)	30.32 (2.40)	0.97
**Nasal width**	36.85 (3.16)	32.87 (3.29)	<0.0001*

*Significant at p < 0.05.

When the sexes were analyzed separately, a similar result was observed. In both men and women, the thirds of the face were also found to significantly differ from each other (both *p* < 0.0001). The orbital (EFL and ICD) and orbito-nasal canons (ICD and NW) also significantly differed from each other (both *p* < 0.0001).

The only sex differences in the measurements in our study were in the lower third of the face (*sn-gn*) and nasal base (*al-al*), with males exhibiting a larger lower third and nasal width than females.

Pairwise comparison indicated that the mean difference was significantly different from 0 for all paired measurements, except the upper and lower thirds of the face, which did not significantly differ among men (*p* = 0.7261). In Saudi men, the lower third was larger than the middle third by a mean difference of 10.9 mm (Table 2).

**Table 2:** **The mean difference in measurements between the vertical, orbital, and orbito-nasal canons as a pairwise comparison**

**Canon**	**Males**	***p*** **value**	**Females**	***p*** **value**
**Vertical Canons**				
Upper third – middle third *tr-n = n-sn*	10.6	<0.0001*	12.3	<0.0001 *
Middle third – lower third *n-sn = sn-gn*	−10.9	<0.0001*	−7.1	<0.0001*
Upper third – lower third *tr-n = sn-gn*	−0.3	<0.0001*	5.2	0.7261
**Orbital canon**				
Eye fissure length –intercanthal distance *ex-en = en-en*	−2.8	<0.0001*	−2.0	<0.0001*
**Orbito-nasal canon**				
Intercanthal distance –Nasal width *en-en = al-al*	−7.1	< 0.0001*	−2.3	< 0.0001*

* *Statistically significant difference between the measurements.*

Variation from the neo-facial canons is shown in Table 3. The vertical canons and the orbital canon demonstrated no sexual dimorphism. However, a sex difference was observed in the orbito-nasal canon. None of the tested neoclassical facial canons were valid for the majority of the sample population. In men, the most frequently occurring neoclassical canon was the orbital canon, observed in 29% of the participants, and the least frequently observed was the equal length of the middle and lower thirds, seen in only 5.4% of the participants. In women, the most frequently met neoclassical canon was the orbito-nasal canon, in 33.3% of participants, whereas the least frequent was the equal length of the upper and middle thirds, seen in only 9.3% of the subjects.

**Table 3:** **Comparison of neo-canons and their variation according to sex by a difference of more than or less than 1 mm**

**Canon**	**Males N = 75**	**Females N = 93**	***p*** **value**
Vertical Canon			
*tr-n = n-sn*	9.7	9.3	0.9398
***tr-n > n-sn***	**81.7**	**88**	
*tr-n < n-sn*	8.6	2.7	
*n-sn = sn-gn*	5.4	10.7	0.2020
*n-sn > sn-gn*	3.2	1.3	
***n-sn < sn-gn***	**91.4**	**88**	
*tr-n = sn-gn*	21.5	14.7	0.2560
***tr-n > sn-gn***	**39.8**	**72**	
*tr-n < sn-gn*	38.7	13.3	
Orbital Canon			
*ex-en = en-en*	29.0	25.0	0.5930
***ex-en > en-en***	**55.9**	**74.7**	
*ex-en < en-en*	15.1	17.3	
Orbito-Nasal Canon			
*en-en = al-al*	6.5	33.3	<.0001*
*en-en > al-al*	1.1	10.7	
***en-en < al-al***	**92.4**	**56.0**	

***Bold font*** *denotes the most predominant variation of the canons in both sexes.*

* *Statistically significant at p < 0.05.*

Overall, the majority of males and females demonstrated a larger upper than middle third, a larger lower than middle third, a larger upper than lower third, a wider EFL length than ICD, and a predominately wider NW than ICD.

## Discussion

The objective of esthetic and reconstructive surgery is to restore ideal and acceptable facial proportions with respect to the ethnic background of the individual. In orthognathic surgery, where osteotomies of the maxilla and mandible are performed to establish dental and facial balance and harmony, facial proportions serve as a guide to the movement of the maxilla-mandibular complex. Maxillofacial surgeons are always on a quest to find more objective guides to facial harmony and balance.

Farkas and his co-workers are credited for extensive work in recording anthropometric facial measurements of healthy individuals from different ethnic backgrounds. These studies have revealed the variability of facial proportional relationships [10,11,20-22].

Based on the work of Farkas and others, facial anthropometric findings in healthy North American white, Chinese, and African American populations have indicated that the neoclassical facial canons were not valid [11]. Farkas found the equal facial thirds originally described by Albrecht Dürer in the 1500s [2,10] to be present only in a small percentage of African and white Americans [10,11,20].

In one multi-center study published in 2005, Farkas et al. assessed 14 anthropometric measurements in 1470 healthy individuals drawn from five regions of the world, 53.1% of whom were of Caucasian origin and the remainder from 13 countries in Europe and three countries in the Middle East (Egypt, Iran, and Turkey) [16]. To our knowledge, that report is the only large-scale study of these anthropometric measurements in subjects from the Middle East. However, these “Middle Eastern” ethnicities are different from that of the Arabian Peninsula, which has distinct and unique facial features. A summary of the results of the current study, compared to those in other Saudi samples, Arab samples, and other ethnicities is shown in Table 4.

**Table 4:** **Comparison between the mean anthropometric facial measurements in the current study and other studies of Arab populations and other ethnicities**

**Ethnic group N**			**Upper Third**		**Middle third**		**Lower third**		**EFL**		**ICD**		**NB**	
			**tr-n**		**n-sn**		**sn-gn**		**en-ex**		**ex-ex**		**al-al**	
			**M**	**F**	**M**	**F**	**M**	**F**	**M**	**F**	**M**	**F**	**M**	**F**
**Current Study**	Saudi	M = 75 F = 93	64.7 (7.5)	65.5 (6.9)	54.1 (4.3)	53.2 (4.1)	65.0 (5.2)	60.3 (4.6)	32.9 (2.7)	32.4 (3.4)	30.3 (3.1)	30.3 (2.4)	36.6 (3.2)	32.9 (3.3)
**Dharap et al. 2013**	Gulf Arabs	M = 51 F = 117	N/A	N/A	N/A	N/A	N/A	N/A	N/A	N/A	N/A	N/A	37.1 (3.4)	33.2 (2.4)
**Algaidi et al. 2012**	Egyptian	M = 108 F = 88	N/A	N/A	54.7 (0.5)	45.8 (0.2)*	63.8 (0.6)*	56.4 (0.6)*	N/A	N/A	N/A	N/A	39.8 (0.4)*	32.8 (0.5)
**Bokhari 2011**	Saudi	M = 276 F = 392	N/A	N/A	N/A	N/A	N/A	N/A	30.8 (2.9)*	29.5 (2.8)*	32.7 (2.8)*	31.3 (3.5)*	N/A	N/A
**Husein et al. 2010**	Indian American	F = 102	N/A	63.9 (7.4)	N/A	58.1 (5.5)*	N/A	57.8 (7.5)*	N/A	30.6 (2.4)*	N/A	31.2 (3.7)*	N/A	35.6 (3.3)*
**Farkas et al. 2005**	Egyptian	M = 30 F = 30	63.6 (9.5)	61.2 (6.0)*	54.6 (5.6)	47.4 (5.2)*	64.1 (6.4)	57.8 (4.5)*	31.5 (1.8)*	30.8 (1.8)*	31.8 (2.1)*	30.9 (2.3)	32.4 (4.0)*	29.3 (3.7)*
**Farkas et al. 2005**	Iranian	M = 30 F = 30	53.4 (8.2)*	56.9 (8.3)*	62.6 (3.2)*	66.2 (4.4)*	73.3 (4.3)*	66.2 (4.4)*	37.2 (3.5)*	24.4 (3.3)*	27.3 (2.7)*	24.6 (3.5)*	35.3 (3.0)	32.1 (2.5)
**Farkas et al. 2005**	Turkish	M = 30 F = 30	61.9 (6.1)	60.7 (7.0)*	58.1 (3.5)*	55.2 (4.0)*	65.9 (4.2)	59.1 (3.8)	30.6 (1.2)*	29.8 (1.6)*	32.8 (2.6)*	31.7 (2.2)*	36.8 (2.3)	32.9 (2.1)
**Farkas 2005**	North American white	M = 109 F = 200	67.1 (7.5)*	63.0 (6.0)*	54.8 (3.3)	50.6 (3.1)*	72.6 (4.5)*	64.3 (4.0)*	31.3 (1.2)*	30.7 (1.2)*	33.3 (2.7)*	31.8 (2.3)*	34.9 (2.1)*	31.4 (2.0)*
**Farkas et al. 2005**	Chinese	M = 30 F = 30	67.1 (6.9)	64.1 (7.5)	53.5 (2.8)	51.7 (3.3)	72.7 (5.2)*	66.4 (5.6)*	29.4 (1.2)*	28.5 (1.8)*	37.9 (3.3)*	36.5 (3.2)*	39.2 (2.9)*	37.2 (2.1)*
**Farkas et al. 2007**	African American	M = 50 F = 50	72.0 (7.8)*	67.1 (5.9)	51.8 (3.1)*	48.8 (3.7)*	78.7 (7.3)*	71.5 (5.2)*	32.9 (1.7)	32.4 (2.4)	35.8 (2.9)*	34.4 (3.4)*	44.1 (3.4)*	40.1 (3.2)*

Values are expressed as mean measurements and standard deviation in parenthesis. EFL: eye fissure length, ICD: intercanthal distance, NB: nasal base. *Statistically significant at p < 0.05.

With regard to the Saudi population, very few studies have addressed anthropometric facial measurement. In 2011, Bukhari [23] assessed four anthropometric eye measurements in Saudi men and women. In comparison to our results, the previously reported EFL was lower and ICD was higher (both statistically significant at *p* <0.05). The difference between our study and Bukhari’s results might be due to the different sample populations. Our study cohort consisted of young adults, while their subjects ranged from 15 to over 70 years old.

Another study that assessed anthropometric measurements of the Saudi Arabian nose was conducted by Al-Qattan et al. [24], who reported a higher ICD and lower NW in both sexes and a shorter middle third of the face in women in comparison to our study. However, due to differences in measurement technique, with the previous study using the indirect anthropometry method of photogrammetry, the results of the two studies cannot be directly compared.

The mean nasal width of an Arab cohort from the Gulf region measured in a study by Dharap et al. [25] is consistent with that of our study, although the term “Arabian Gulf” was used loosely by the authors, since the subjects were from five countries of the Gulf Cooperation Council. The origin of people from the Gulf is not uniform; it can be Persian, Turkish, East Asian, or other.

Anthropometric studies in regions such as the Gulf are very difficult because of the possible influence of inter-racial marriage and immigration on anthropometric facial measurements. The term “Saudi” is a nationality and not an ethnicity. Saudi Arabia is located in the Arabian Peninsula, and although most Saudis are ethnically Arab, originating from tribes in the Arabian Peninsula, people of the Kingdom of Saudi Arabia are mixed and can be descended from Iran, Turkey, East Asia, Russia, and Africa. We were unable to find a previous study involving a cohort of young Saudi Arabian adults originating purely from the Arabian Peninsula. Additionally, none of the studies involving Saudi populations observed or tested the validity of the neoclassical facial canons. Therefore, the present study tested the validity of the neo-classical canons on young adults from the Arabian Peninsula, with the aim to define a cohort of individuals with the same ethnic origins.

The only sex differences observed among the measurements in this study were the longer lower facial third and NW demonstrated by men. The majority of the population, both men and women, had longer upper and lower thirds than middle third, which is consistent with the findings of Farkas et al. that North American white and African American faces exhibited a larger chin than the canonical face [10,11].

The validity of the vertical canon of equal facial thirds was not confirmed in our sample. The middle third was the smallest of the three, and the majority of participants (91.4% of men and 88% of women) demonstrated a larger lower third than middle third. This observation is also consistent with the findings of Farkas et al. (2000), where the middle third was the smallest third in both North American whites and African Americans. In that study, the lower third of the face was larger than the middle third in 100% of both populations, and the upper third was larger than the middle third in 100% of African American and 95% of North American white participants [11].

Farkas et al. [11] also found the orbital canon (palpebral fissure length equal to ICD) to be valid in only 33% of North American white and 13% of African American participants. The ICD was predominately wider than EFL in African Americans (73%). The opposite was true in our study population, in whom the majority (55.9% of men and 74.7% of women) had wider EFL than ICD. Thus, those individuals in our study with valid orbital canons (25% of women and 29% of men) fall between African Americans and North American whites. By contrast, in a Chinese Han population, Dawaei et al. found that the most predominant variation of the orbital canon was a wider intercanthal distance than eye fissure length. The measurements were equal in about 35.5 percent of the Chinese Han population [20].

The most frequently validated canon among those tested was the nasoorbital canon (NW equal to ICD) in females (33.3%), but this canon was valid in only 6.5% of men. This finding is in contrast to the results of Farkas et al. (1985), where 40.8% of North American whites and 3% of African Americans demonstrated a valid orbito-nasal canon [10]. In Chinese subjects, an equal nasoorbital canon was found in approximately one-third of the population studied (35.4%) [20].

The most predominant variation in our sample was a wider NW than ICD (56% in females and 92% in males). Similarly, this was the most predominant variation of the nasoorbital canon in African Americans (94%), whereas in North American whites, the wider nose was seen in 27.9% of participants [10,11].

## Conclusions

To date, no studies testing the validity of the neo-classical facial cannons originally described by artists of the Renaissance and adopted by plastic, maxillofacial, and reconstructive surgeons in an ethnically homogenous Saudi population. Our study sample was carefully selected to consist of young adults originating only from the Arabian Peninsula. In general, we observed a trend for a longer lower third in comparison to middle third of the face, a wider eye fissure length than intercanthal distance, and a wider nasal base in comparison to intercanthal distance. Men had a significantly larger lower third and nasal width measurement than women.

The neoclassical facial canons could not be validated in this cohort of young adults originating from the Arabian Peninsula. Like any ethnicity, facial norms and measurements of other Caucasian populations cannot be applied to the Arab population. This distinction has a great impact on treatment planning for corrective, reconstructive, and orthognathic procedures. Further studies in our region should aim at establishing baseline values for all the facial anthropometric measurements recommended by Farkas and his colleagues among people from the Arabian Peninsula.

## Abbreviations

ICD: Intercanthal distance (*en-en*)
EFL: Eye fissure length (*ex-en*)
NW: Nasal width (*al-al*)
